# A qualitative study of the experiences of pregnant women in accessing healthcare services during the Zika virus epidemic in Villavicencio, Colombia, 2015–2016

**DOI:** 10.1002/ijgo.13045

**Published:** 2020-01-23

**Authors:** Hector M. Gomez, Carlos Mejia Arbelaez, Jovana A. Ocampo Cañas

**Affiliations:** ^1^ Public Health, Medical Education, and Professionalism School of Medicine University of los Andes Bogotá Colombia

**Keywords:** Access, Colombia, Experience, Healthcare services, Pregnant women, Qualitative, Zika virus

## Abstract

**Objective:**

To explore the perceptions and experiences of pregnant women in accessing healthcare services during the epidemic in Colombia during 2015–2016.

**Methods:**

A qualitative study using semistructured interviews was conducted in Villavicencio. Six women who had been diagnosed with Zika virus infection during their pregnancies and whose fetus had suspected microcephaly participated in the investigation. Grounded theory was used and thematic content analysis was made for each category identified.

**Results:**

Three main themes affecting access to healthcare services were identified: (1) women knew basic information about the virus, but it was limited; (2) access to services was delayed due to their lack of availability or limited supply in the municipality; and (3) most of the participants made out‐of‐pocket payments to get access to services that were not provided.

**Conclusions:**

Several gaps were identified in the provision of healthcare services to pregnant women during the Zika epidemic. Policy makers need to utilize the results from affected communities to develop and implement public policies that adapt and respond to their priorities and needs.

## Abstract 2

Policy makers need to consider identified gaps when they adapt and implement new clinical care guidelines for maternal and perinatal health.

## 1 INTRODUCTION

A year after the 2015 Zika virus outbreak in Latin America, the World Health Organization (WHO) recognized a causal relationship between Zika virus infection during pregnancy and the development of newborn microcephaly and neurologic sequelae.^1^, ^2^, ^3^ Before this outbreak, the known signs and symptoms of Zika virus infection had only included mild fever, malaise, headache, arthralgia, conjunctivitis, and rash.^4^ Although there were reports of Guillain‐Barré syndrome associated with Zika virus,^5^ no deaths had been reported.^6^ Given the neurologic sequelae in newborns associated with Zika virus infection during pregnancy, the high direct and indirect costs related to microcephaly management and consequences,^7^ and the risk of future outbreaks, Zika virus has since been considered a public health priority.^8^

In the Americas, from October 25, 2015 to December 29, 2016, there were 177 614 confirmed cases of Zika virus infection and 2525 confirmed congenital syndromes associated with it.^9^ In Colombia, there were 6363 confirmed cases of Zika virus infection in pregnant women since the beginning of the epidemic in 2015 through December 31, 2016.^10^ Moreover, there were 318 cases of newborn microcephaly attributed to pregnancy‐related Zika virus infection during the epidemic phase of the virus through July 2016.^11^

The department of Meta, with a population of 961 292, was fourth among the departments in Colombia that registered the most confirmed cases of pregnancy‐related Zika virus infections (8.7%), behind Norte de Santander (16.8%), Valle del Cauca (12.9%), and Huila (10.8%).^10^ Meta contributed to the overall national burden, with around 9% of confirmed infections and 2% of newborns with microcephaly.

In response to the 2015 epidemic, WHO raised a call to action for local governments to develop public health interventions that would aim to prevent the consequences of Zika virus infection, particularly in pregnant women and women of childbearing age. Following these recommendations, the Colombian Ministry of Health (MoH) and the National Institute of Health (NIH) developed a series of guidelines and protocols to mitigate the effects of the outbreak.^12^, ^13^ While guidelines for the general population were published in January 2016, it was not until February 2016 that a guideline specifically addressed the clinical approach for pregnant women and women of childbearing age at risk of Zika virus infection.^14^ This document was intended to give healthcare providers evidence‐based information about prevention strategies, counseling for women and their families, diagnosis criteria, and other measures including guidance on voluntary termination of pregnancy and follow‐up of suspected and confirmed cases to mitigate the consequences of exposure to Zika virus during pregnancy.

Despite the existence of these national guidelines since February 2016, additional confirmed cases of pregnant women infected with Zika virus and newborns afflicted with microcephaly arose after this date. Previous studies of vector‐borne diseases explained an association between poor health outcomes and some determinants of health.^15^, ^16^ Specifically for Zika virus, factors that can affect the prevention, diagnosis, and management of the infection and its consequences include poor vector control strategies, poor sanitation, knowledge gaps in Zika transmission and its health consequences, lack of strategies for birth control, and poor access to healthcare services such as prenatal care.^17^, ^18^, ^19^ To our knowledge, there are no studies that explore factors that influence access to healthcare services from the perspective of pregnant women infected with Zika virus. Recognizing and understanding the experiences of women in accessing health care during the Zika virus outbreak will help improve the guidelines and their implementation in future outbreaks.

The aim of the present study was to explore and analyze the experiences in accessing healthcare services of pregnant women diagnosed with Zika virus whose newborns had microcephaly.

## 2 MATERIALS AND METHODS

The study was conducted in the municipality of Villavicencio, Meta, Colombia between September 1, 2015 and December 31, 2016. Even though Villavicencio only contributed a small proportion of the disease burden, we chose this municipality because it had a strong surveillance system and rapidly implemented the WHO, Colombian MoH, and Colombian NHI protocols.

We conducted a qualitative study using semistructured interviews of pregnant or recently pregnant women infected with Zika virus. The study was a subcomponent of a larger project looking at the health system response and clinical approaches to pregnant women affected by Zika virus in two municipalities of Colombia.

Study participants were purposely selected from the National Public Health Surveillance System (SIVIGILA) and the municipality's records. A total of 12 cases of microcephaly associated with Zika virus infection in the mother during the perinatal period were identified and selected as the study population. Of these 12 cases, five had incomplete identification and contact information, wrong phone numbers, or did not answer phone calls despite several attempts, which excluded them from the study. One participant was excluded because she had moved to another city. The authors contacted the remaining six eligible participants via telephone to provide a general description of the project including background, objectives, and methods.

A total of six semistructured interviews were conducted using a research instrument that had been previously piloted, adjusted, and refined based on the women's feedback, the conceptual framework chosen for this research, and the aim of the study. The interview guide included sociodemographic characteristics, followed by guiding questions around two main topics. The first set of questions focused on maternity care and family planning, which encompassed basic knowledge about family planning methods and its usage history, maternity expectations, and cultural beliefs. The second set of questions focused on healthcare services, with an in‐depth inquiry regarding the step‐by‐step process of accessing services, as well as knowledge about prevention and transmission of Zika virus, counseling on voluntary termination of pregnancy, pregnancy outcomes, follow‐up performed during their pregnancy and after delivery, and overall satisfaction with healthcare services. The complete set of themes, subthemes, and guiding questions can be found in supporting information Table S1.

One of the authors (HG) conducted all interviews in Spanish between April 20, 2018 and June 20, 2018. Each of the interviews lasted between 21 and 60 minutes. They were performed in private to ensure confidentiality at a location chosen by the participant. In some cases, and by request of the participant, relatives accompanied and contributed to the interview.

The authors obtained written informed consent before each interview. All interviews were audio recorded. The authors reassured the participants that they did not have any legal affiliations with healthcare providers or insurers and that participation in the study would not affect the care they had been receiving in any way. Participants could withdraw consent at any point during the research and they could avoid answering any question they did not want to answer.

Data were analyzed using grounded theory, where the researcher generates a theory with regard to a phenomenon, process, or action that comes from a specific context and from the perspective of different participants. Those theories should derive from data that were obtained during field exploration and should be compared with prior literature. Additionally, we used the conceptual framework of access to healthcare services proposed by Levesque et al.^20^ This framework defines access to healthcare as the opportunity to identify healthcare needs, to seek, reach, obtain, or use healthcare services, and to have those needs fulfilled.^20^

Interviews were transcribed verbatim and cross‐checked by the authors for accuracy. Furthermore, the interviewer included field notes in each transcript that provided complementary information regarding the context and behaviors of the interviewee that could not be captured in the audio recordings.

During the analysis phase, a set of categories were defined based on the instrument guide and the conceptual framework chosen for the analysis. A deductive thematic analysis was used to elaborate a codebook with the definition of each category and subcategory, to provide the coder with a guide for the encoding process. These category definitions were based on the conceptual framework, existing literature, and in concepts and procedures outlined in the national management guidelines for women suspected to have Zika virus infection. Besides the previously described deductive approach, the authors implemented an inductive approach to code qualitative data, allowing the coding of new emergent categories while the text was examined. Next, review and re‐evaluation of the obtained categories and subcategories was made to simplify and refine them according to the conceptual framework initially proposed. A final set of categories and subcategories was achieved. The detailed set of categories can be found in supporting information Table S2. NVivo 12 (QSR International, Doncaster, Vic., Australia) was used for codification of data.

This research was executed according to one of the objectives included in the larger project. Ethical approval for the larger project was obtained from the University of los Andes’ Ethics Review Committee (certificate No. 658, 2016) and by the PAHO Ethics Review Committee on July 20, 2017 (certificate No. PAHO‐2017‐04‐0042).

## 3 RESULTS

A total of six women participated in the study. The average age of the participants was 26 years (range, 21–30 years). At the time of the interview, two participants had a professional degree, three were in university, and one had completed high school. Five participants were in stable relationships, and one was a single mother. Four were permanent residents of Villavicencio, and two lived in nearby municipalities. Three women were primiparous, and the rest had had one prior pregnancy. All participants referred to their pregnancies as wanted, but only half referred to it as a planned pregnancy.^21^

Three main themes were identified: (1) Zika virus knowledge; (2) availability of healthcare resources and timely access to services; and (3) out‐of‐pocket payments for access to healthcare services.

### 3.1 Zika virus knowledge

#### 3.1.1 What did women know about Zika virus and the epidemic in their town?

By the time of pregnancy, all women reported having a basic knowledge of Zika virus infection such as signs and symptoms, transmission, prevention strategies, and the possible consequences for the fetus. All participants knew mosquitoes transmitted the virus and that Zika could cause a disease. When asked about other ways of transmission, only one participant mentioned vertical transmission and sexual intercourse.

Regarding signs and symptoms, participants recognized fever, malaise, and cutaneous rash as the most common clinical manifestations. One of the participants referred that the symptoms of Zika virus infection were similar to those of the Chikungunya virus.

Women talked about how to reduce the risk of Zika virus infection. Strategies included using insect repellent and bed nets, as well as impacting the life cycle of the mosquito by avoiding stagnant water deposits at home, as well as fumigation. Furthermore, two women mentioned postponing pregnancy during an epidemic to reduce the risk of the fetus being affected by Zika virus.

All women understood that Zika virus infection during pregnancy could have consequences for the fetus. While some referred to the consequences as general severe defects, most mentioned microcephaly as a significant sequela in the baby.

All participants knew they were at risk of Zika virus infection in their municipality given the geographic characteristics of the municipality and the epidemic situation.

#### 3.1.2 Where did the participants obtain information on Zika virus?

All women revealed that they acquired most of the information about Zika virus from the news, other media, family members, and friends. Although all participants attended prenatal checkups with nurses, general practitioners, or gynecologists, five women did not receive any information regarding Zika virus risks, modes of transmission, or the risks of sequelae for their babies. Only one participant reported that she had been actively reminded during prenatal visits to her physician about ways to reduce the risks of transmission and possible consequences for her baby. Two of the participants reported that they sought advice and help from physicians when they thought they had been infected by the Zika virus.

I was almost five months pregnant, and then they [physicians] said that nothing was going to happen to my baby since the baby was already formed. Participant 3, age 23

Right now Zika is trending. In a few months another disease will come out, let's say “the AH1N1: next year that disease is going to come out,” he said. […] “Some diseases are coming out, and these are like strategies that medicine has created. They create the disease in order to generate a necessity for such drugs.” Participant 5, age 30

### 3.2 Availability of healthcare resources and timely access to services

#### 3.2.1 Were the required healthcare resources available?

Every participant was able to attend prenatal checkups in Villavicencio during their pregnancy through public health insurance, performed either by a nurse or general practitioner; only a few had access to a gynecologist. One woman said she had to move from her hometown to Villavicencio to access health care and prenatal checkups. However, most women mentioned that prenatal ultrasounds or lab tests were not always possible owing to lack of authorization from their public healthcare provider and lack of timely access to specialists.

All women who were suspected of having Zika virus infection during pregnancy underwent blood tests to confirm the diagnosis. However, samples were transported to Bogota (120 km away) because the necessary infrastructure for sample processing was not available in Villavicencio.

#### 3.2.2 Were women able to access healthcare services on time?

Women explained that prenatal checkups, laboratory tests, and obstetric ultrasounds were not always provided promptly by their public healthcare insurance. One participant mentioned she received timely health care because she had access to private providers.

One of the most common themes throughout the interviews was the significant delay and the difficulty in obtaining test results that had been performed to confirm or rule out Zika virus infection. Some women mentioned that they had pursued legal actions to obtain these results. One woman received the results of her confirmatory test when her baby was 5 months old although the sample had been taken during her seventh week of pregnancy. Similarly, another woman stated:

We went every eight days there, where I had the tests, to find out when we were going to obtain them. However, the answer from them was that it was taking longer because the tests were not performed here in Villavicencio. So, the test results took around one year. Participant 1, age 22

### 3.3 Out‐of‐pocket payments to access healthcare services

Most women reported that they made direct out‐of‐pocket payments to healthcare providers during their pregnancy or during childbirth to access specialty care, laboratories, or special procedures. This was because these services were either not covered by their public healthcare insurance or because they were not provided on time. One woman mentioned that she had to pay out‐of‐pocket for ultrasounds during pregnancy.

## 4 DISCUSSION

All participants knew something about Zika virus; however, there were some important knowledge gaps, especially surrounding the asymptomatic course of Zika virus. This viral infection is asymptomatic in up to 80% of cases.^22^ Despite the high level of education of participants, another knowledge gap was recognition of transmission other than mosquito bites.

Most participants revealed that by the time they were pregnant they knew of the possible neurologic sequelae for their developing fetus if they became infected during pregnancy. This information is crucial for all women of childbearing age and pregnant women since knowing the possible consequences for their child can improve adherence to prevention strategies and follow‐up care during pregnancy. A population that recognizes the risk of infection can help shape health behaviors and improve health outcomes,^23^ which can facilitate future public health interventions.

Notwithstanding the national guidelines provided by the Colombian MoH that healthcare providers should share Zika virus information with women at risk of infection,^14^ only one of the participants mentioned that she was actively reminded of this information at every prenatal checkup. The remaining women made it clear that they were not counseled about the virus. Instead, most of the information came from TV news, radio, and the internet. Even though education provided by healthcare professionals needs to be improved, policymakers could also take advantage of the grand scope of the media and consider this strategy for disseminating public health knowledge to the population.^24^

Accurate evidence‐based information was not always provided to women at risk. This is critical given that the association between Zika virus infection in pregnant women and neurologic consequences for their fetuses had been widely recognized by 2016.^25^, ^26^, ^27^, ^28^, ^29^, ^30^ On the other hand, some women have received incorrect information questioning the reality of the epidemic and the origin of the disease.^31^, ^32^ This lack of accurate information provided by healthcare professionals can have a severe impact on diagnosis and treatment of patients seeking medical advice. Further investigation needs to focus on health professionals’ knowledge and communication strategies about Zika virus, and how patient education is being disseminated.

Despite the availability of specialists in obstetrics and gynecology in Villavicencio, prenatal checkups were predominantly carried out by nurses or general practitioners for women suspected to have Zika virus infection during their pregnancy. According to national guidelines, these women should be followed as a high‐risk pregnancy, hence, requiring care by specialists. Lack of knowledge of the existing guidelines during pregnancy could have negatively impacted the follow‐up by specialists. In addition, lack of follow‐up could have resulted from administrative barriers associated with a woman's Administrative Entity of Benefits Plan (EAPB, Spanish acronym).

Women reported delayed access to prenatal laboratory testing and ultrasounds, including test results, owing to administrative reasons such as delayed authorization from their EAPB. Another factor identified in the interviews was the fragmentation of the public health system in which a lack of coordination between stakeholders influences how services are provided.^33^ Other factors contributing to delays in obtaining test results were samples processed only in the capital city, high demand for the service nationwide, and no clarity in protocols for the delivery of results to women—all leading to lack of coordination between the NIH, EAPBs, local health authorities, and hospitals.

Delay in diagnosis can have severe consequences for affected women, limiting actions during the pregnancy such as seeking early fetal confirmation tests or voluntary termination of pregnancy, which—according to national guidelines—has to be counseled to affected women given the risk of severe neurologic sequelae for their children.^14^

Access to healthcare services for newborns with suspected microcephaly associated with Zika virus was difficult for most of the women interviewed mainly due to the limited number of required specialized physicians in Villavicencio, which resulted in delayed medical appointments. According to the national guidelines for the clinical management of congenital anomalies of fetuses exposed to Zika virus during pregnancy, a thorough and comprehensive follow‐up is required^34^; however, most women agreed that their children did not get the appropriate health care promptly. This calls into question the appropriate interpretation and implementation of the guidelines by healthcare providers; furthermore, that the guidelines may be omitting the context in which the epidemic happened given the lack of availability of laboratories, procedures, and specialized physicians in the affected municipalities.

Health authorities in the municipality of Villavicencio, and other regions in Colombia with similar contexts, need to take into consideration the gaps in healthcare services that have been identified in the present study when they adapt and implement new clinical care guidelines (such as PAIS [Healthcare Attention Policies], MIAS [Integral Healthcare Attention Model], and RIAS [Integral Healthcare Attention Routes]) for maternal and perinatal health.^35^, ^36^

All women in the study made out‐of‐pocket payments either during their pregnancy or for their affected child. These payments were made to access medical consults, laboratory tests, and procedures that are included in the Obligatory Health Plan (POS, Spanish acronym). They were not performed for several reasons that acted as barriers, including multiple administrative procedures and authorization denials by a woman's EAPB. In other cases, women made out‐of‐pocket payments to access procedures in a timely manner.

Out‐of‐pocket expenditure should not occur for provision of these services since they are included in the POS, and hence are covered by the public healthcare insurer. Provision of a sufficient supply of services according to the population's needs, reducing administrative barriers for access to services, and determining why out‐of‐pocket payments are being made need to be investigated. Evidence shows that higher out‐of‐pocket payments result in poorer population health outcomes.^37^

The study has some limitations. The participants selected were cases reported to SIVIGILA; therefore, the experiences of women not recorded in the public surveillance system were not included in this study. Moreover, from the 12 cases identified in the surveillance system, we were only able to interview six women because there was erroneous or missing contact information. This reflects the need to improve the quality of reporting undertaken by healthcare professionals through the notification sheets. The possible consequences of not including the entire eligible population include the risk of leaving out different experiences than those reported in the study, and consequently variation in the overall results.

The study was conducted in only one of the affected municipalities during the epidemic in Colombia. For a broader approach in other contexts, experiences of women with the same characteristics are being investigated in the larger project—of which the present study forms a part—in other affected municipalities in Colombia. Considering the specific localization of the present study, transferability of the results to other municipalities or environments should be considered with caution.

In terms of policy recommendations from the study, health policies to address and mitigate Zika virus infection must consider the particularities of the virus and how it affects women.

Basic knowledge of modes of transmission, prevention strategies, signs and symptoms, and possible neurologic consequences for the fetus need to be maintained and expanded not only for pregnant women but also for women of reproductive age and their partners; taking advantage of the media as the main source of information recognized in the study should be considered. For this purpose, an intersectoral approach is needed, including stakeholders involved at all levels—from health authorities and policymakers to the affected community.

Prioritized attention should be given to pregnant women including raising their awareness of the signs and symptoms of the virus, actions to prevent infection, possible consequences for their developing fetus, and counseling about their right to access voluntary termination of pregnancy when applicable.

Sexual and reproductive health education should focus on women aged between 15 and 49 years, as well as for men with a female partner of reproductive age. Furthermore, it should be included during preconception and prenatal checkup counseling on aspects such as the effects of Zika virus infection, different methods used for prevention, diagnosis, and management. This education should be provided by the corresponding healthcare professional who is following the pregnancy.

Providing healthcare services on time could reduce the out‐of‐pocket payments revealed by women in the present study since most of these costs were due to the delay in accessing those services. Availability of timely healthcare services can be achieved by guaranteeing sufficient human resources, which should include gynecologists‐obstetricians, pediatricians, and neuropediatricians.

The lack of continuity of care highlights the lack of coordination and the fragmentation of the healthcare system and the poor communication among stakeholders. Improved collaborative work and close follow‐up of these patients will positively impact their health outcomes.

Increased surveillance by health authorities, such as the national superintendence of health, of EAPBs and institutions providing healthcare services is needed to guarantee that the national guidelines for the management and treatment of pregnant women and children affected by Zika virus are accurately implemented and followed. One way to encourage adherence to guidelines is a recognition and reward system for EAPBs and healthcare services institutions such as certifications or distinctions of national recognition for excellence in maternal–infant services.

In conclusion, access to healthcare services by pregnant women with suspected Zika virus infection whose fetuses had suspected or confirmed microcephaly was positively influenced by high levels of knowledge of basic information about Zika virus and negatively influenced by poor provision of timely healthcare, lack of continuity in health care, and out‐of‐pocket expenditure. These gaps and perceived barriers to access were in part due to the fragmentation of the healthcare system and lack of interaction and communication between the different stakeholders.

We encourage investigators to conduct further qualitative research that explores access to healthcare services for women affected by Zika virus. Understanding the population's needs and priorities, and identifying the gaps in healthcare services, can lead to improvements in the provision of services for current or future affected populations.

## AUTHOR CONTRIBUTIONS

JAOC and HG designed the study and collected the data. HG analyzed and interpreted the data. HG, JAOC, and CMA contributed to manuscript preparation. The authors are responsible for the opinions expressed in this publication, which do not necessarily reflect the opinions or political decisions of the institutions to which they are affiliated, or of the institutions who kindly supported the study.

## CONFLICTS OF INTEREST

The authors have no conflicts of interest.

## Supporting information


**Table S1.** Themes, subthemes, and corresponding guiding questions. Translated from Spanish.
**Table S2.** Summary of principal categories, subcategories, definition, and total number of references coded.
