# Supplementary material for: A qualitative study of the experiences of pregnant women in accessing healthcare services during the Zika virus epidemic in Villavicencio, Colombia, 2015–2016
Source: Int J Gynaecol Obstet. 2020 Jan 23;148(Suppl 2):29–35. doi: 10.1002/ijgo.13045 (PMC7065052; doi:10.1002/ijgo.13045)
Supplement: Supplementary file 1 — Table S1. Themes, subthemes, and corresponding guiding questions. Translated from Spanish. Table S2. Summary of principal categories, subcategories, definition, and total number of references coded. [file IJGO-148-29-s001.docx]

**Supporting information S1.** Themes, subthemes, and corresponding guiding questions. Translated from Spanish.

| Themes | Subthemes | Guiding questions |
| --- | --- | --- |
| Maternity and family planning | Maternity expectations and beliefs | What does maternity mean to you? Did you want or plan to have kids? |
|  | Family planning | What do you know about contraceptive methods? What did you think about family planning? What does your partner say in regard to family planning? What contraceptive methods did you use? How was the experience? |
| Healthcare services | Zika virus | What do you know about Zika? Where did you hear that information from? What ways of transmission can you identify? |
|  |  |  |
|  | Infection counseling | How did you know you were infected by Zika virus? Did you seek health care? What did healthcare professionals tell you about Zika? |
|  | Congenital defect diagnosis | How was the experience of the diagnosis process of the alteration that your child had in the head by the doctor and the healthcare system overall? Did you receive any type of counseling from the healthcare providers in regard to your child's alteration? |
|  | Voluntary termination of pregnancy (VTP) | What do you know about VTP? Did someone inform you about it before or during pregnancy? |
|  | Psychosocial support | Did you have emotional support throughout the process from the moment of your child's diagnosis until now? Who provided it? |
|  | Healthcare satisfaction | How did you feel cared for by the doctors and the healthcare provider during your pregnancy and after your child was born? Is there anything that can be done to improve it? |
|  | Follow-up | Do you think that you had the necessary controls and counseling from healthcare providers throughout the pregnancy and after your child was born? |

**Supporting information S2.** Summary of principal categories, subcategories, definition, and total number of references coded.

| Categories | Subcategories | | Definition/use | Number of references |
| --- | --- | --- | --- | --- |
|  |  |  |  |  |
| Maternity | Expectations and beliefs | | What does motherhood mean to the woman, what were the expectations once they became mothers? Feelings about pregnancy. | 7 |
|  | Pregnancy planning | | Was the pregnancy wanted/planned? | 7 |
| Ability to perceive [1] | Health knowledge | | “Denotes facts, information, and skills acquired through experience or education, as well as the theoretical or practical understanding of a subject related to health and health care” [2]. | 111 |
|  |  | Communication by physician | Physicians provide information about Zika risks/consequences during appointments. | 18 |
|  |  | Family planning | Knowledge about family planning methods. | 16 |
|  |  | Sources of information | Where did the information they knew about health come from? | 27 |
|  |  | Voluntary termination of pregnancy | Knowledge about VTP, what was it, were they informed about the possibility? | 14 |
|  |  | Zika virus infection | Basic knowledge about definition, transmission, prevention strategies, signs/symptoms, association with birth defects. | 28 |
|  | Trust in health care | | Did women trust in the healthcare system? Did they trust in information or treatments provided by physicians/nurses? | 11 |
| Ability to seek [1] | Autonomy | | Were women autonomous in decision-making throughout their pregnancy, were the decisions influenced by partner/family/social values. | 3 |
|  | Gender bias | | Prejudices or preconceptions from healthcare providers and partners toward female gender influencing different aspects of her life. | 5 |
| Ability to reach [1] | Healthcare resources availability | | Availability of healthcare services including hospitals, specialized physicians, procedures, etc. | 13 |
|  | Job flexibility | | “Characteristics of individuals (e.g. duration and flexibility of working hours) in order to attend or look for health care” [1] | 2 |
|  | Services provided in a timely manner | | Possibility for the client to obtain the services they require without delays that put his life or health at risk [3]. | 34 |
| Ability to pay [1] | Out of pocket payments | | Payments made by individuals to healthcare providers at the time of service use. | 25 |
|  | Private health insurance | | Use of private health insurance to access healthcare services. | 8 |
| Ability to engage [1] | Empowerment | | “Process through which individuals and social groups are able to express their needs, present their concerns, devise strategies for involvement in decision-making, and achieve political, social, and cultural action to meet those needs” [4]. | 17 |
|  | Quality of interpersonal relationship | | How was the interaction between women and healthcare professionals, how they felt about it? | 18 |
| Healthcare consequences | Perception of healthcare services | | How was the overall perception of healthcare services throughout the pregnancy? | 35 |
|  | Quality of life impact | | How did her life change or was impacted by her pregnancy outcome? | 13 |

**References**

[1] Levesque J-F, Harris MF, Russell G. Patient-centred access to health care: conceptualising access at the interface of health systems and populations. *Int J Equity Health*. 2013;12:18.

[2] Weingarten SR, Stone E, Green A, et al. A study of patient satisfaction and adherence to preventive care practice guidelines. *Am J Med*. 1995;99:590-596.

[3] Bakar ZA, Fahrni ML, Khan TM. Patient satisfaction and medication adherence assessment amongst patients at the diabetes medication therapy adherence clinic. *Diabetes Metab Syndr Clin Res Rev*. 2016;10:S139-S143.

[4] Trzeciak S, Gaughan JP, Bosire J, Mazzarelli AJ. Association Between Medicare Summary Star Ratings for Patient Experience and Clinical Outcomes in US Hospitals. *J Patient Exp*. 2016;3:6-9.
